# Effects of functional training on linear sprint and change-of-direction performance in soccer players: a systematic review and meta-analysis

**DOI:** 10.3389/fphys.2026.1951674

**Published:** 2026-09-29

**Authors:** Hongyu Yang, Ya Lan, Yu Chen, Shang Zeng

**Affiliations:** 1 School of Football, Chengdu Sport University, Chengdu, Sichuan, China; 2 School of Physical Education, Chengdu Sport University, Chengdu, Sichuan, China

**Keywords:** change of direction, functional training, linear sprint, meta-analysis, physical performance, soccer, systematic review

## Abstract

**Background:**

Functional training is increasingly incorporated into soccer conditioning; however, its effects on linear sprint and change-of-direction (COD) performance remain unclear. This systematic review and meta-analysis evaluated the effects of structured functional training on linear sprint and directional-change performance in soccer players.

**Methods:**

PubMed, Web of Science, Embase, SPORTDiscus via EBSCOhost, Scopus, and the Cochrane Library were searched from database inception to 6 July 2026. Randomized controlled trials comparing structured functional training with usual soccer training, no additional training, or other non-functional training conditions were included. Pooled effects were calculated as Hedges’ g with 95% confidence intervals (CIs) using random-effects models. Exploratory subgroup analyses were conducted according to intervention duration, weekly training frequency, and sprint distance. The certainty of evidence was assessed using the Grading of Recommendations Assessment, Development and Evaluation (GRADE) approach.

**Results:**

Ten randomized controlled trials involving 260 soccer players were included. Functional training significantly improved pre-planned COD performance (nine trials; n = 240; Hedges’ g = −0.72, 95% CI −1.23 to −0.22; p = 0.005; I^2^ = 71%) and linear sprint performance (seven trials; n = 164; Hedges’ g = −1.00, 95% CI −1.54 to −0.46; p = 0.0003; I^2^ = 61%). Leave-one-out sensitivity analyses showed that the direction and statistical significance of both pooled effects were retained after exclusion of individual studies, although the magnitude of the COD and sprint estimates was influenced by Zhao et al. and Doungchan et al., respectively. No statistically significant between-subgroup differences were identified according to intervention duration, weekly training frequency, or sprint distance.

**Conclusion:**

Structured functional training may improve linear sprint and pre-planned COD performance in soccer players. However, the magnitude of the pooled effects should be interpreted cautiously because of between-study heterogeneity, the influence of individual studies, and the limited evidence base. Current evidence is insufficient to establish an optimal training prescription or to extrapolate these findings to reactive agility or match performance.

**Systematic review registration:**

https://www.crd.york.ac.uk/PR, identifier CRD420261441220.

## 1 Introduction

Modern soccer is characterized by frequent transitions and repeated high-intensity actions, including accelerations, sprints, decelerations, directional changes, turns, physical duels, and subsequent reaccelerations. Linear sprint and change-of-direction (COD) abilities are therefore important physical qualities underpinning rapid offensive and defensive transitions, separation from opponents, defensive recovery, and the creation of attacking space. Although soccer performance depends on the integrated contribution of endurance, speed, strength, power, and agility, short-distance sprinting and COD performance are widely regarded as key indicators of soccer-specific physical capacity (Little and Williams, 2005; Stolen et al., 2005). Accordingly, identifying effective training strategies to improve these qualities remains an important objective in soccer conditioning and performance research.

Despite their shared dependence on speed-related capabilities, linear sprinting and COD performance represent related but distinct physical constructs. Linear sprint performance primarily reflects the ability to accelerate and attain high running speeds along a straight path and is commonly evaluated using 10-, 20-, or 30-m sprint tests. Haugen et al. highlighted that short-duration sprinting occurs frequently during soccer match play and that sprint performance may distinguish players competing at different performance levels (Haugen et al., 2014). Similarly, Little and Williams reported that acceleration, maximal running speed, and agility were correlated but not interchangeable in professional soccer players, indicating a degree of specificity among these speed-related qualities (Little and Williams, 2005). A systematic review of speed-testing procedures in soccer further demonstrated that the interpretation of sprint performance depends partly on the distance and protocol used (Altmann et al., 2019). These findings suggest that training-induced changes in linear sprint performance should be interpreted in relation to the specific distance assessed and the acceleration or speed qualities represented by that distance.

COD performance places additional demands on braking ability, postural control, and neuromuscular coordination. To execute a rapid directional change, an athlete must effectively decelerate, lower and reposition the center of mass, redirect momentum, and subsequently reaccelerate in a new direction. Performance in these tasks is influenced not only by linear speed but also by lower-limb strength and power, eccentric braking capacity, trunk stability, dynamic balance, coordination, movement technique, and neuromuscular control. Importantly, COD performance should be distinguished from agility. Sheppard and Young defined agility as a rapid whole-body movement involving a change in velocity or direction in response to an external stimulus (Sheppard and Young, 2006). Young et al. subsequently emphasized that pre-planned COD speed and reactive agility are associated but distinct constructs and should therefore be differentiated in both training and assessment (Young et al., 2015). Previous reviews have also indicated that COD performance is determined by an interaction among strength, speed, braking capacity, technique, and neuromuscular characteristics and may respond differently to different training modalities (Nygaard Falch et al., 2019). Accordingly, the present review distinguishes pre-planned COD performance from reactive agility according to whether the task involves a predetermined movement path or an externally triggered perceptual or decision-making response. Reactive agility was not conceptually excluded from the review scope; however, the eligible evidence ultimately identified was limited to pre-planned COD tasks.

Functional training has increasingly been incorporated into athletic conditioning programs. However, it does not represent a single, universally standardized training modality, and substantial variation exists in how the term is applied across the literature. Interventions described as functional training may emphasize different combinations of multi-joint and multiplanar movement, core stability, dynamic balance, neuromuscular coordination, strength and power development, and sport-related movement patterns (Kibler et al., 2006; La Scala Teixeira et al., 2017; Xiao et al., 2021; Luo et al., 2022). Therefore, the present review adopted a review-specific operational definition to support reproducible study selection and to distinguish functional training from conventional strength, power, plyometric, sprint, and other conditioning approaches. This operational definition was intended specifically for the purposes of the present review rather than as a universal definition of functional training.

Previous intervention studies have suggested that functional training may improve speed-, power-, and COD-related performance across athletic populations, including soccer players (Sander et al., 2013; Alonso-Fernández et al., 2017; Usgu et al., 2020; Ambuja and Lokendra Bahadur, 2021). However, the magnitude and consistency of these adaptations remain uncertain, particularly when functional training is considered as a distinct conditioning approach in soccer.

Nevertheless, findings across individual studies of functional training remain inconsistent. Some trials have reported favorable changes in sprint- or COD-related performance, whereas others have found limited or non-significant effects (Turna and Alp, 2020; Gürgen and Kilinç Boz, 2023). Such variation may reflect differences in participant characteristics, intervention content and progression, comparator conditions, training duration and frequency, and outcome-assessment procedures. Considerable variation also exists in the tests used to quantify performance, including sprint tests over different distances and pre-planned COD tasks with different movement patterns, turning angles, and technical demands. These differences complicate direct comparisons across individual trials and support the need for quantitative synthesis.

Importantly, sprint and COD adaptations in soccer players have already been quantitatively examined under related conditioning paradigms. For example, meta-analytic evidence has evaluated speed, agility, and quickness training (Sun et al., 2025), as well as strength and plyometric training (Pardos-Mainer et al., 2021). These reviews demonstrate that sprint and COD performance can be modified through several conditioning approaches, but they do not specifically address structured functional training as operationalized in the present review. Previous reviews of functional training have instead examined heterogeneous athletic populations and broader physical-performance outcomes (Bashir et al., 2022). To our knowledge, no previous meta-analysis has quantitatively synthesized randomized controlled trials specifically examining the effects of structured functional training, as defined in the present review, on linear sprint and pre-planned COD performance in soccer players. Furthermore, although intervention duration, training frequency, and related training characteristics have been examined in adjacent conditioning literature (Pardos-Mainer et al., 2021), it remains unclear whether these factors modify the effects of functional training specifically in soccer players.

Therefore, this systematic review and meta-analysis aimed to quantify the effects of structured functional training on linear sprint and directional-change or agility-related performance in soccer players and to explore whether intervention duration, weekly training frequency, and sprint distance were associated with variation in the observed effects.

## 2 Materials and methods

### 2.1 Study design and registration

This systematic review and meta-analysis synthesized evidence from randomized controlled trials examining the effects of functional training on linear sprint and directional-change or agility-related performance in soccer players. The review was reported in accordance with the Preferred Reporting Items for Systematic Reviews and Meta-Analyses (PRISMA) 2020 statement (Page et al., 2021), and the completed PRISMA 2020 checklist is provided in **Supplementary Table S1**. The review protocol was prospectively registered in the International Prospective Register of Systematic Reviews (PROSPERO; CRD420261441220) on 4 July 2026, before the formal database searches were conducted.

### 2.2 Search strategy

A systematic literature search was conducted in six electronic databases: PubMed, Web of Science Core Collection, Embase, SPORTDiscus via EBSCOhost, Scopus, and the Cochrane Library. Each database was searched from inception to 6 July 2026. The search strategy combined terms relating to three core concepts: functional training, soccer players, and physical-performance outcomes. Terms related to functional training included “functional training,” “functional strength training,” “functional exercise,” “core stability training,” “suspension training,” “TRX,” and “bodyweight training.” Soccer-related terms included “soccer,” “football,” “soccer player,” and “football player,” whereas outcome-related terms included “sprint,” “linear sprint,” “speed,” “acceleration,” “change of direction,” “change-of-direction,” “COD,” and “agility.” Search terms were combined using the Boolean operators AND and OR and were adapted to the indexing conventions, controlled vocabulary, and search fields of each database. The principal database search strategies are presented in **Table 1**, and the complete reproducible search strategies for all databases are provided in **Supplementary Table S2**.

**Table 1:** Detailed search strategies from selected databases.

Database	Search strategy
PubMed	(“functional training” OR “functional strength training” OR “functional exercise” OR “core stability training” OR “suspension training” OR TRX OR “bodyweight training”) AND (“soccer” OR “football” OR “soccer players” OR “football players”) AND (“sprint” OR “linear sprint” OR “speed” OR “acceleration” OR “change of direction” OR “change-of-direction” OR COD OR “agility”)
Web of Science	TS = (“functional training” OR “functional strength training” OR “core stability training” OR “suspension training” OR TRX OR “bodyweight training”) AND (“soccer” OR “football” OR “soccer players” OR “football players”) AND (“sprint” OR “linear sprint” OR “speed” OR “change of direction” OR “change-of-direction” OR COD OR “agility”)
Embase	(‘functional training’ OR ‘functional strength training’ OR ‘functional exercise’ OR ‘core stability training’ OR ‘suspension training’ OR TRX OR ‘bodyweight training’) AND (‘soccer’ OR ‘football’ OR ‘soccer player’ OR ‘football player’) AND (‘sprint’ OR ‘linear sprint’ OR ‘speed’ OR ‘acceleration’ OR ‘change of direction’ OR ‘change-of-direction’ OR COD OR ‘agility’)
EBSCO	(“functional training” OR “functional strength training” OR “functional exercise” OR “core stability training” OR “suspension training” OR TRX OR “bodyweight training”) AND (“soccer” OR “football” OR “soccer players” OR “football players”) AND (“sprint” OR “linear sprint” OR “speed” OR “acceleration” OR “change of direction” OR “change-of-direction” OR COD OR “agility”)
Scopus	TITLE-ABS-KEY (“functional training” OR “functional strength training” OR “functional exercise” OR “core stability training” OR “suspension training” OR TRX OR “bodyweight training”) AND (“soccer” OR “football” OR “soccer players” OR “football players”) AND (“sprint” OR “linear sprint” OR “speed” OR “acceleration” OR “change of direction” OR “change-of-direction” OR COD OR “agility”)
Cochrane Library	(“functional training” OR “functional strength training” OR “functional exercise” OR “core stability training” OR “suspension training” OR TRX OR “bodyweight training”) AND (“soccer” OR “football” OR “soccer players” OR “football players”) AND (“sprint” OR “linear sprint” OR “speed” OR “acceleration” OR “change of direction” OR “change-of-direction” OR COD OR “agility”)

To identify additional eligible studies, the reference lists of all included articles and relevant systematic reviews were screened manually. No restrictions were imposed on publication year. Only full-text articles published in English were eligible for inclusion.

### 2.3 Inclusion and exclusion criteria

Eligibility criteria were established using the Population, Intervention, Comparator, Outcomes, and Study Design (PICOS) framework and are summarized in **Table 2**. Because functional training is not a single, universally standardized training modality, interventions were classified using a review-specific, content-based operational definition rather than solely according to the terminology used by the original authors or the equipment employed. This operational definition was informed by previous conceptual literature and systematic reviews of functional training in athletic populations (Kibler et al., 2006; Xiao et al., 2021; Bashir et al., 2022).

**Table 2:** Selection criteria used in the meta-analysis.

Category	Inclusion criteria	Exclusion criteria
Population	Male and female soccer players of any age and competitive level, including youth, adolescent, amateur,collegiate,semi-professional and professional players.	Non-soccer players; athletes from other sports; injured players undergoing rehabilitation; patients with clinical conditions; mixed-sport samples in which soccer players could not be separately extracted.
Intervention	Structured functional training interventions performed over multiple training sessions, including functional strength training, core stability training, suspension training, bodyweight training, neuromuscular functional training, or soccer-specific functional training programs.	Acute warm-up protocols only; single-session interventions; injury rehabilitation programs; interventions not primarily based on functional training; studies in which the effects of functional training could not be isolated.
Comparator	Active or passive control conditions, including regular soccer training, usual training, no additional training, traditional strength training, conventional conditioning, or alternative training without functional training components.	Studies without a control group; single-group pre–post studies; studies comparing two functional training programs only; studies in which the comparator included functional training components.
Outcomes	At least one measure of linear sprint performance or change-of-direction/agility performance, such as 5-m, 10-m, 20-m or 30-m sprint tests, 505 test, T-test, Illinois Agility Test, Arrowhead Agility Test, shuttle run, zigzag test, or other validated COD/agility tests.	Studies not reporting linear sprint, sprint speed, acceleration, change-of-direction or agility outcomes; studies reporting only strength, balance, jump, endurance, body composition, technical skills or injury outcomes without relevant performance data.
Study design	Randomized controlled trials or randomized intervention studies with pre- and post-intervention measurements.	Non-randomized controlled trials, quasi-experimental studies without randomization, cross-sectional studies, observational studies, case studies, conference abstracts, reviews, editorials, protocols, theses and dissertations.

For the purposes of this review, functional training was operationalized at the intervention-program level. An eligible intervention was required to consist of a structured, multi-session training program in which the principal training content emphasized integrated multi-joint and multi-segment movement patterns rather than predominantly isolated muscle actions. Such programs incorporated broader movement-integration characteristics, including multiplanar or unilateral movements, dynamic postural control, balance or proprioceptive demands, trunk–lower-limb coordination, neuromuscular or kinetic-chain integration, acceleration and deceleration, or task- or sport-related movement patterns. Functional training was required to constitute the principal or independently identifiable training component so that its effects could be distinguished from those of concurrently applied training modalities.

Classification was based on the characteristics of the overall intervention rather than on the use of any specific exercise or equipment. Accordingly, the use of suspension systems (e.g., TRX), bodyweight exercises, free weights, resistance machines, elastic resistance, unstable surfaces, or plyometric exercises was neither sufficient nor, by itself, exclusionary for classification as functional training. Similarly, conventional resistance, strength, power, sprint, or plyometric programs were not considered functional training solely because they involved sport-relevant movements; they were eligible only when the overall intervention satisfied the integrated movement characteristics described above.

Studies involving male or female soccer players of any age or competitive level were eligible. Eligible interventions were structured, multi-session programs meeting the operational definition of functional training, with usual soccer training, no additional training, or other non-functional conditioning programs serving as comparators. Eligible outcomes included linear sprint and directional-change or agility-related performance. Directional-change outcomes were classified as pre-planned COD or reactive agility according to whether the task involved a predetermined movement path or an externally triggered perceptual or decision-making response. Only randomized controlled trials providing sufficient data for effect-size calculation were included; non-soccer populations, rehabilitation samples, ineligible interventions, insufficient outcome data, and non-randomized studies were excluded.

### 2.4 Selection process

All records retrieved from the electronic database searches were imported into EndNote X9 (Clarivate Analytics, Philadelphia, PA, USA) for reference management. Duplicate records were removed using the automated duplicate-detection function in EndNote, followed by manual verification.

After deduplication, two reviewers (HY and SZ) independently screened the remaining records in two stages. First, titles and abstracts were assessed against the prespecified eligibility criteria. Records were excluded at this stage when they clearly involved non-soccer populations, interventions unrelated to functional training, outcomes unrelated to linear sprint or change-of-direction (COD) performance, ineligible study designs, or non-eligible publication types such as reviews, conference abstracts, study protocols, theses, and dissertations. Records for which eligibility could not be determined from the title and abstract were retained for full-text assessment.

In the second stage, the same two reviewers independently assessed the full texts of all potentially eligible reports according to the prespecified PICOS criteria. Eligibility was determined by examining the participant characteristics, intervention content, comparator condition, outcome measures, study design, and availability of sufficient data for quantitative synthesis. The specific reason for exclusion was recorded for each report excluded after full-text assessment. Disagreements between the two reviewers were resolved through discussion and consensus; when consensus could not be reached, a third reviewer adjudicated the final decision.

### 2.5 Data extraction

Two reviewers independently extracted data from all included studies using a standardized data-extraction form. The extracted data were subsequently cross-checked for accuracy and completeness. Disagreements were resolved through discussion and consensus; when consensus could not be reached, a third reviewer adjudicated the final decision.

The extracted information included study characteristics, participant characteristics, intervention and comparator details, and outcome data. Specifically, the following variables were recorded: first author, publication year, country, study design, sample size, participant age and sex, competitive level, type of functional training, intervention duration, weekly training frequency, session duration, comparator condition, and the means, standard deviations (SDs), and sample sizes for linear sprint and directional-change outcomes.

Linear sprint outcomes included 10-, 20-, and 30-m sprint-test performance. Because the included studies assessed sprint performance over different distances, eligible sprint measures were classified under the broader domain of linear sprint performance and further categorized according to sprint distance. Directional-change outcomes were classified according to the task characteristics rather than solely according to the terminology used by the original authors. Tests involving a predetermined movement path without an external stimulus or decision-making requirement, including the 505 test, T-test, T-drill, Arrowhead test, zig-zag test, and shuttle-run test, were classified as pre-planned change-of-direction (COD) outcomes. Tests requiring participants to perceive and respond to an external visual, auditory, opponent-based, or other unpredictable stimulus were classified as reactive-agility outcomes. Although reactive-agility outcomes were eligible for extraction, no included study ultimately reported a reactive-agility measure; therefore, all directional-change outcomes available for quantitative synthesis represented pre-planned COD performance.

Change-score data were extracted preferentially. When mean change scores were not reported directly, they were calculated by subtracting the baseline value from the post-intervention value. When the SD of the change score was unavailable, it was estimated from the reported baseline and post-intervention SDs using the approach described in the data-synthesis section. For time-based outcomes, lower values indicated better performance; therefore, effect-size directions were harmonized so that negative values favored functional training. For multi-arm trials in which more than one intervention arm met the eligibility criteria, all eligible intervention arms were extracted and subsequently combined, where appropriate, before effect-size calculation to avoid double-counting a shared comparator. When a study reported multiple eligible sprint distances or multiple directional-change tests, all relevant outcome data were extracted and classified according to their respective outcome domains before application of the outcome-selection and quantitative-synthesis procedures described below.

### 2.6 Risk of bias assessment

Risk of bias was assessed using the revised Cochrane Risk of Bias tool for randomized trials (RoB 2; version 22 August 2019), with the effect of assignment to intervention as the target estimand. Because RoB 2 is result-specific, assessments were conducted separately for linear sprint and pre-planned change-of-direction (COD) outcomes, where applicable. Five domains were evaluated: bias arising from the randomization process, bias due to deviations from intended interventions, bias due to missing outcome data, bias in measurement of the outcome, and bias in selection of the reported result. The RoB 2 signaling questions and decision algorithms were used to derive domain-level and overall judgments of low risk of bias, some concerns, or high risk of bias. Two reviewers independently assessed each eligible outcome result, and disagreements were resolved through discussion and consensus; when necessary, a third reviewer adjudicated the final judgment.

Because blinding of participants and intervention providers is generally not feasible in exercise-training trials, lack of blinding was not considered an independent reason for automatically assigning a high risk of bias. Instead, the potential influence of awareness of group allocation was considered within the domain addressing deviations from intended interventions. Similarly, whether knowledge of group allocation could have influenced test administration or outcome assessment was considered within the outcome-measurement domain. Risk-of-bias assessments were summarized using separate outcome-specific traffic-light plots for linear sprint and COD performance, with detailed domain-level judgments and supporting rationales provided in **Supplementary Table S4**.

Although the PROSPERO record listed both the original Cochrane Risk of Bias tool and RoB 2 as possible approaches, RoB 2 was selected as the sole risk-of-bias framework for the final review. This clarification represents a refinement of the registered methodological description rather than the use of two parallel risk-of-bias frameworks.

The certainty of evidence for each primary outcome was assessed using the Grading of Recommendations Assessment, Development and Evaluation (GRADE) approach. Evidence from randomized controlled trials was initially rated as high certainty and was subsequently downgraded, where appropriate, for risk of bias, inconsistency, indirectness, imprecision, and publication bias. The overall certainty of evidence was classified as high, moderate, low, or very low. Two reviewers independently performed the GRADE assessments, with disagreements resolved through discussion or consultation with a third reviewer. The GRADE assessments are presented in **Supplementary Table S3**.

### 2.7 Data synthesis and statistical analysis

Separate meta-analyses were conducted for linear sprint and pre-planned change-of-direction (COD) performance. Although reactive-agility outcomes were eligible, no included study reported an externally triggered reactive-agility measure; therefore, the directional-change synthesis was limited to pre-planned COD performance.

Because sprint outcomes were assessed over different distances and COD performance was evaluated using heterogeneous test protocols, effect estimates were expressed as standardized mean differences and corrected for small-sample bias using Hedges’ g, with corresponding 95% confidence intervals (CIs).

Change scores were used for quantitative synthesis. When mean changes were not reported directly, they were calculated as the post-intervention mean minus the baseline mean. Directly reported change-score standard deviations (SDs) were used preferentially. When unavailable, change-score SDs were estimated as:


$$SD_{change}=\sqrt{SD_{pre}^{2}+SD_{post}^{2}-2r\left(SD_{pre}\right)\left(SD_{post}\right)}$$

assuming a pre–post correlation of (r = 0.80). For time-based outcomes, negative effect estimates indicated greater reductions in completion time and therefore favored functional training.

For multi-arm trials with more than one eligible intervention arm, eligible intervention groups were combined before effect-size calculation to avoid double-counting a shared comparator. In Fayazmilani et al., the TRX and bodyweight-training arms were combined into a single functional-training group.

For the primary sprint analysis, one eligible sprint outcome per study was included. No *a priori* hierarchy was specified for selecting among multiple eligible sprint distances reported within the same study. Erol et al. was the only study reporting both eligible 10-m and 30-m sprint outcomes. The 30-m outcome used in the original primary analysis was retained, and an outcome-selection sensitivity analysis was conducted by replacing it with the corresponding 10-m outcome while keeping all other study data unchanged.

Random-effects meta-analyses were conducted using the inverse-variance method with the DerSimonian–Laird estimator for between-study variance. Heterogeneity was evaluated using Cochran’s Q, (τ^2^), and (I^2^). Primary and subgroup meta-analyses were conducted in Review Manager version 5.3, whereas sensitivity and small-study-effect analyses were conducted in Stata version 17.0. All statistical tests were two-sided, with p< 0.05 considered statistically significant.

### 2.8 Subgroup and sensitivity analyses

Exploratory subgroup analyses were conducted according to sprint distance (20 vs. 30 m), intervention duration (≤6 vs. ≥8 weeks), and weekly training frequency (≥3 vs. <3 sessions per week) (McKay et al., 2022). The sprint-distance analysis was performed to explore whether the estimated effects of functional training differed according to the distance used to assess linear sprint performance. The intervention-duration and training-frequency analyses were conducted to examine whether these training-dose characteristics potentially moderated the effects of functional training.

Subgroup analyses were conducted only when at least two studies were available within each subgroup. Between-subgroup differences were evaluated using formal tests for subgroup interaction. Given the limited number of included studies and the small number of studies within several subgroups, all subgroup analyses were considered exploratory and hypothesis-generating. Accordingly, non-significant subgroup interactions were not interpreted as evidence that the effects were equivalent across training conditions or participant groups.

Leave-one-out sensitivity analyses were performed by sequentially excluding each individual study and recalculating the pooled effect estimate. The robustness of the findings was evaluated by examining whether the direction, magnitude, confidence interval, or statistical significance of the pooled effect changed materially after the exclusion of any single study.

### 2.9 Publication bias

Potential small-study effects were explored using visual inspection of funnel plots, Egger’s regression test, and Begg’s rank-correlation test. Funnel-plot asymmetry and statistically significant Egger’s or Begg’s tests were considered possible indications of small-study effects rather than definitive evidence of publication bias. The findings were interpreted in conjunction with the magnitude of between-study heterogeneity, the results of sensitivity analyses, and the methodological characteristics of the included studies. Because fewer than 10 studies contributed to each primary outcome, these analyses were considered exploratory and interpreted cautiously owing to the limited power of funnel plots and statistical tests to detect asymmetry.

## 3 Results

The electronic database searches identified 742 records: 103 from PubMed, 256 from Web of Science, 25 from Embase, 68 from SPORTDiscus via EBSCOhost, 51 from Scopus, and 239 from the Cochrane Library. An additional eight records were identified through manual screening of the reference lists of included studies and relevant reviews. After removing 219 duplicate records, 531 unique records remained for title and abstract screening.

Of these, 495 records were excluded because they clearly did not meet the eligibility criteria, primarily owing to an ineligible population, an intervention that could not be classified as functional training, an ineligible study design, or the absence of relevant linear sprint or change-of-direction (COD) outcomes. The full texts of 36 potentially eligible reports were subsequently assessed. Twenty-six reports were excluded at the full-text stage for reasons including the absence of an eligible comparator, insufficient data for effect-size calculation, an intervention that did not meet the operational definition of functional training, an ineligible athletic population, or the absence of an eligible outcome. Ultimately, 10 randomized controlled trials were included in the systematic review and meta-analysis. The study-selection process is presented in the PRISMA flow diagram (**Figure 1**).

**Figure 1:**
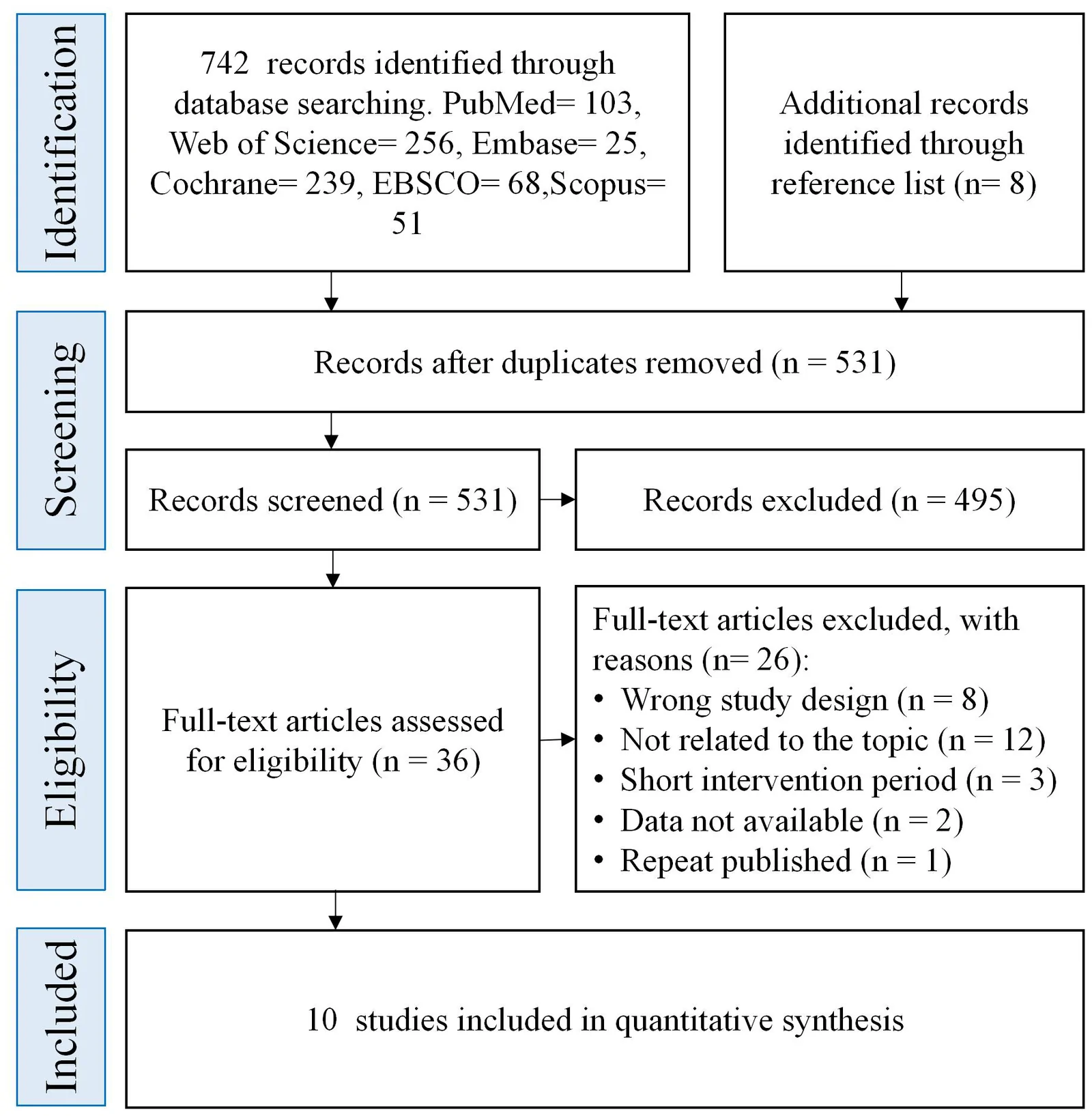
PRISMA flow diagram of the study selection process.

### 3.1 Study characteristics

Ten randomized controlled trials involving a total of 260 soccer players were included (Turna and Alp, 2020; Fayazmilani et al., 2022; Philip et al., 2022; Erol et al., 2023; Gürgen and Kilinç Boz, 2023; Atıcı and Bayrakdar, 2025; İleri and Müniroğlu, 2026; Doungchan et al., 2026; Haghighi et al., 2026; Zhao et al., 2026). Participants were predominantly male and ranged in age from approximately 10 to 25 years, spanning youth and adult populations. The included samples comprised youth, amateur, recreational, and professional soccer players. The studies were conducted in Türkiye, Thailand, Iran, India, and China.

The functional training interventions varied considerably across studies and included TRX-based functional training, TRX suspension training, bodyweight training, functional strength training, high-intensity functional training, Functional Movement Screen (FMS)-based functional training, and multicomponent programs integrating strength, agility, and accuracy exercises. Intervention duration ranged from 6 to 12 weeks, with training generally performed two or three times per week. Comparator groups typically continued their usual soccer training, standard soccer drills, or conventional conditioning programs.

Linear sprint performance was assessed using 10-, 20-, and 30-m sprint tests. Pre-planned change-of-direction (COD) performance was evaluated using the Arrowhead test, T-test, zig-zag test, agility shuttle test, COD sprint test, and T-drill. Some studies additionally used soccer-specific movement tasks involving ball control, including a ball-dribbling shuttle run and dribbling around poles. Owing to variation in sprint distance, test configuration, movement demands, and measurement scales, standardized effect sizes were used for quantitative synthesis. The detailed characteristics of the included studies are presented in **Table 3**.

**Table 3:** Characteristics of included studies.

Study	Country	Participants	Design	Intervention	Comparator	Duration / frequency	Relevant outcomes
Atıcı and Bayrakdar, 2025	Türkiye	Youth soccer players, n=19; Exp=10, Con=9; aged 12–15 years	RCT	TRX-based functional training	Regular soccer training	8 weeks; 3 sessions/week; 30 min/session	20m sprint; Arrowhead agility test
Boz 2023	Türkiye	Male football players aged 13–15 years; randomized n=20 (TRX=10, control=10); one control participant withdrew; final analyzed n=19 (TRX=10, control=9)	RCT	TRX exercise program after football training	Routine football training	8 weeks; 2 sessions/week	20-m sprint; Arrowhead agility test
Doungchan et al., 2026	Thailand	Young adult male soccer players, n=30; FTG=15, CG=15; aged 19–24 years	RCT	Functional training including strength, agility and precision-based exercises	Standard soccer drills	6 weeks; 3 sessions/week	30-m sprint; T-test agility
Erol et al., 2023	Türkiye	Male U19 soccer players, n=22; training=11, control=11; mean age ~18 years	RCT	Functional training added to routine football training	Routine football training / foot tennis	6 weeks; 2 sessions/week	10-m sprint; 30-m sprint; zig-zag test
Fayazmilani et al., 2022	Iran	Prepubescent male soccer athletes, n=30; TRX=10, BW=10, control=10; mean age ~12 years	RCT	TRX suspension training or bodyweight training	Regular soccer training only	8 weeks; 2 sessions/week; 45–60 min/session	Agility shuttle test
Haghighi et al., 2026	Iran	Male amateur soccer players, n=24; HIFT=12, CON=12; mean age 24–25 years	RCT	High-intensity functional training replacing part of regular training	Regular soccer training	6 weeks; 3 sessions/week	20-m sprint; change-of-direction sprint
Ileri and Müniroglu, 2026	Türkiye	Male U13–U14 soccer players, n=30; FTG=15, CG=15; mean age: FTG 13.47 y, CG 13.07 y	RCT	Functional strength training added to routine soccer training	Regular soccer training	6 weeks; 3 sessions/week; 30–35 min/session	Sprint and agility tests with/without ball; slalom dribbling
Philip et al., 2022	India	Recreational male soccer players, n=30; experimental=15, control=15; aged 17–25 years	RCT	Functional training plus conventional training	Conventional training only	8 weeks; 3 sessions/week	30-m sprint; T-test agility
Turna and Alp, 2020	Türkiye	Professional male soccer players, n=20; FTG and TTG; mean age ~23–25 years	RCT	Functional training added to soccer training	Traditional soccer training	8 weeks; 2 sessions/week; ~20 min/session	30-m sprint
Zhao et al., 2026	China	Boys aged 10–11 years, n=36; EG=18, CG=18	RCT	FMS-based functional training during warm-up and cool-down	Regular soccer training	12 weeks; 3 sessions/week	T-drill; ball-dribbling shuttle run; dribbling around poles

### 3.2 Risk of bias assessment

Risk-of-bias assessments using RoB 2 are presented separately for linear sprint and pre-planned change-of-direction (COD) outcomes in **Figures 2**, **3**, respectively, with detailed domain-level judgments and supporting rationales provided in **Supplementary Table S4**.

**Figure 2:**
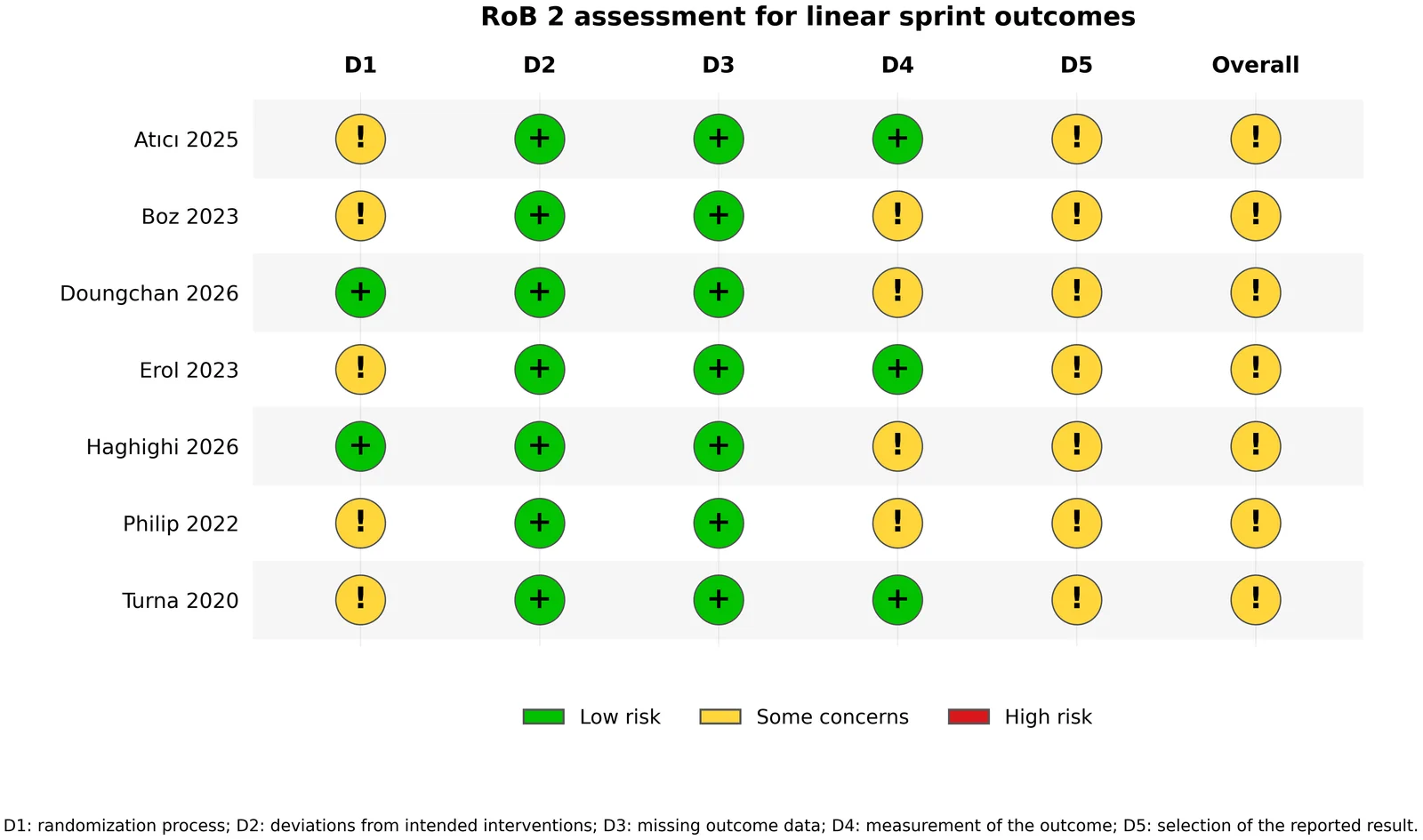
RoB 2 assessment for linear sprint outcomes.

**Figure 3:**
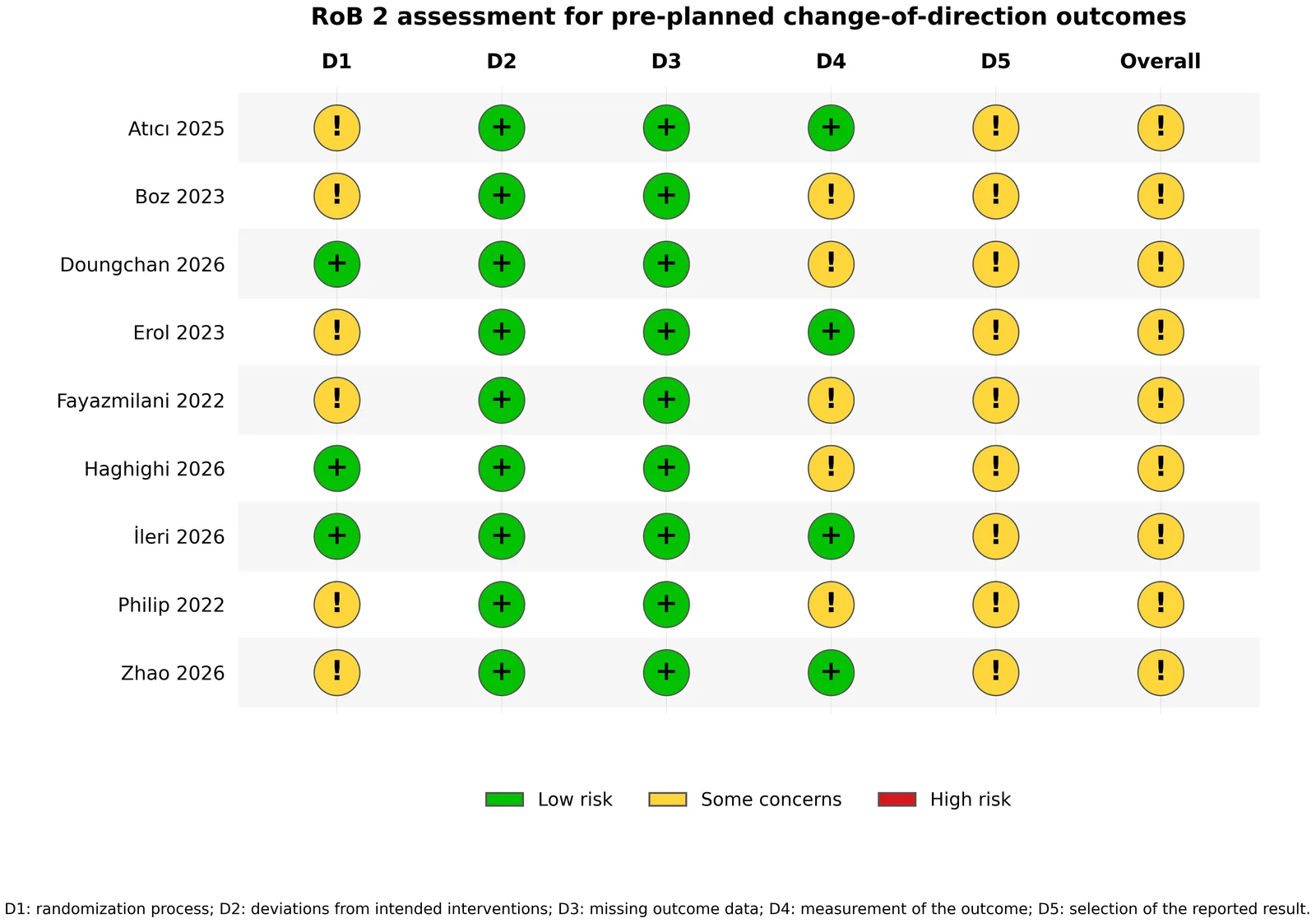
RoB 2 assessment for pre-planned change-of-direction outcomes.

For linear sprint performance, all seven contributing outcome results were judged as having some concerns overall. Concerns primarily arose from limited reporting of the randomization process in several trials, insufficient information regarding outcome-measurement procedures in some studies, and the absence of prospectively available statistical analysis plans. In contrast, concerns related to deviations from the intended interventions and missing outcome data were generally limited.

For pre-planned COD performance, all nine contributing outcome results were also judged as having some concerns overall. Concerns in the measurement-of-outcome domain were more evident in studies that used manual timing or provided insufficient information regarding outcome assessment, whereas studies using electronic timing systems and/or blinded assessors generally had fewer concerns in this domain. As with the sprint outcomes, missing outcome data and deviations from the intended interventions were generally not considered major sources of bias. No outcome result was judged to be at high risk of bias overall.

### 3.3 Effects on pre-planned change-of-direction performance

Nine studies involving 240 soccer players contributed to the meta-analysis of pre-planned change-of-direction (COD) performance, including 126 participants in the functional-training groups and 114 in the comparator groups. As shown in **Figure 4**, the random-effects model showed a significant pooled effect favoring functional training (Hedges’ g = −0.72, 95% CI −1.23 to −0.22; p = 0.005). Because all included outcomes were expressed as task-completion time, negative effect estimates indicated shorter completion times and therefore better pre-planned COD performance in the functional-training groups.

**Figure 4:**
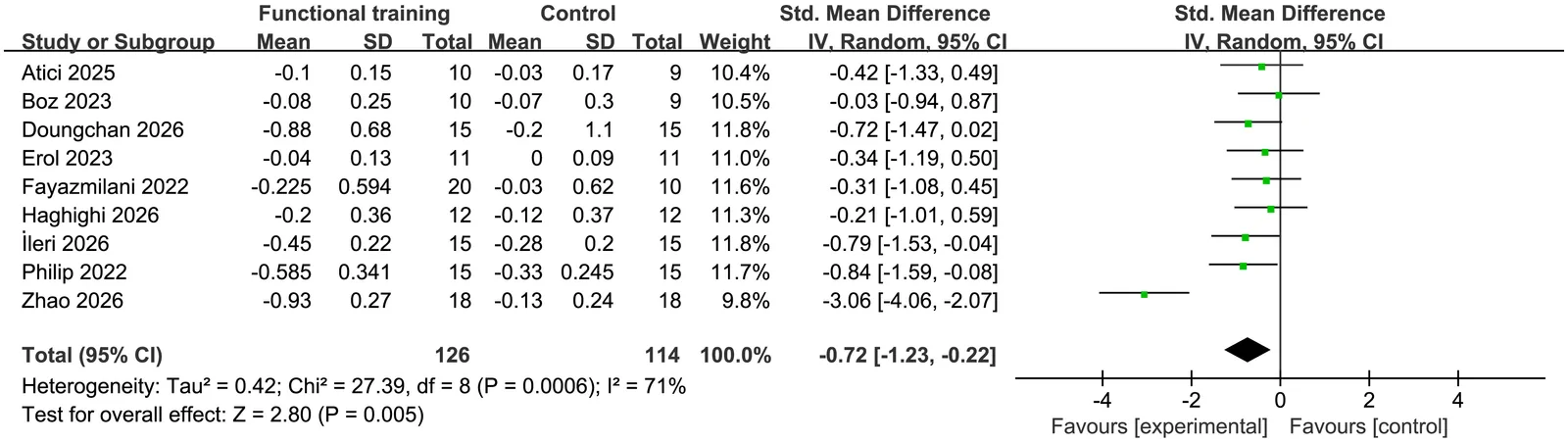
Forest plot of the effect of functional training on pre-planned change-of-direction performance.

Substantial between-study heterogeneity was observed (τ^2^ = 0.42; Q = 27.39, df = 8, p = 0.0006; I^2^ = 71%), indicating considerable variation in the magnitude of the estimated effects across studies.

### 3.4 Effects on linear sprint performance

Seven studies involving 164 soccer players contributed to the meta-analysis of linear sprint performance, including 83 participants in the functional-training groups and 81 in the comparator groups. As shown in **Figure 5**, the random-effects model showed a significant pooled effect favoring functional training (Hedges’ g = −1.00, 95% CI −1.54 to −0.46; p = 0.0003). Because sprint performance was assessed using completion time, negative effect estimates indicated shorter sprint times and therefore better performance in the functional-training groups.

**Figure 5:**
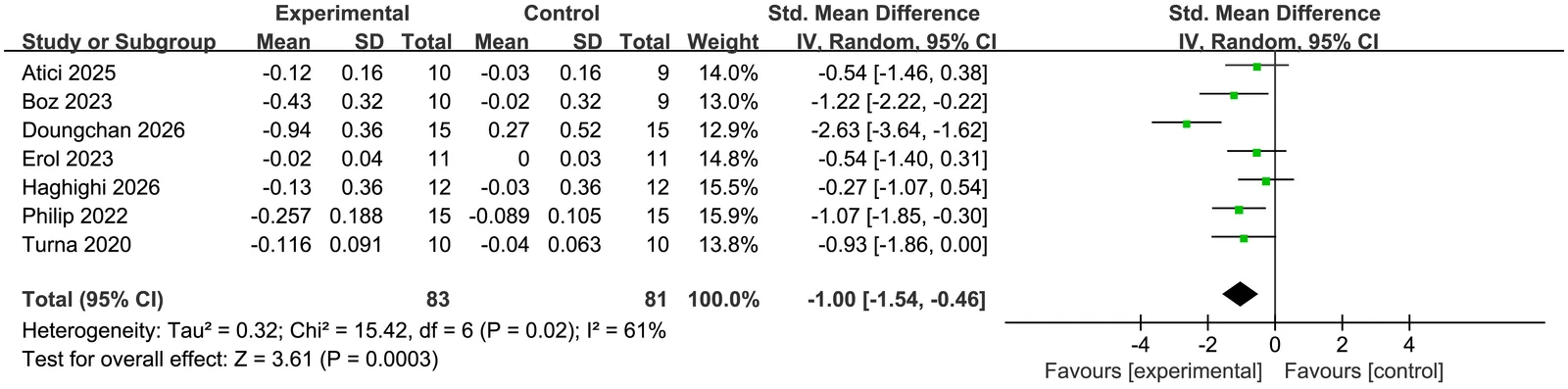
Forest plot of the effect of functional training on linear sprint performance.

Substantial between-study heterogeneity was observed (τ^2^ = 0.32; Q = 15.42, df = 6, p = 0.02; I^2^ = 61%), indicating variation in the magnitude of the estimated effects across studies.

### 3.5 Subgroup analyses

Exploratory subgroup analyses were conducted according to intervention duration, weekly training frequency, and sprint distance. For pre-planned change-of-direction (COD) performance, studies were stratified by intervention duration (≤6 vs. ≥8 weeks) and weekly training frequency (≥3 vs.<3 sessions per week). For linear sprint performance, subgroup analyses were conducted according to intervention duration (≤6 vs. ≥8 weeks), weekly training frequency (≥3 vs.<3 sessions per week), and sprint distance (20 vs. 30 m).

Across most subgroups, the pooled point estimates favored functional training. More clearly favorable estimates were observed for COD performance following interventions lasting ≤6 weeks and programs delivered ≥3 times per week. For linear sprint performance, favorable pooled estimates were observed for interventions lasting ≥8 weeks, programs delivered ≥3 times per week, and 20-m sprint outcomes. However, formal tests for subgroup differences did not provide clear evidence that the effects of functional training varied according to intervention duration, weekly training frequency, or sprint distance.

Given the small number of studies within each subgroup and the resulting statistical uncertainty, these findings should be interpreted as exploratory and hypothesis-generating rather than as evidence of a dose–response relationship or definitive effect modification. Detailed subgroup results are presented in **Tables 4**, **5**, with the corresponding forest plots provided in **Supplementary Figures S1A–E**.

**Table 4:** Subgroup characteristics for feasible subgroup analyses.

Study	Outcome category	Sprint-distance category	Training duration subgroup	Frequency subgroup	Age subgroup	Functional training type
Atıcı and Bayrakdar, 2025	Linear sprint + COD/agility	20 m	≥8 weeks	≥3 sessions/week	Youth	TRX / suspension training
Boz 2023	Linear sprint + COD/agility	20 m	≥8 weeks	<3 sessions/week	Youth	TRX / suspension training
Doungchan et al., 2026	Linear sprint + COD/agility	30 m	≤6 weeks	≥3 sessions/week	Adult	Multicomponent functional training
Erol et al., 2023	Linear sprint + COD/agility	10 m and 30 m	≤6 weeks	<3 sessions/week	Older adolescent / adult	Multicomponent functional training
Fayazmilani et al., 2022	COD/agility only	No linear sprint	≥8 weeks	<3 sessions/week	Youth / prepubescent	TRX / bodyweight training
Haghighi et al., 2026	Linear sprint + COD/agility	20 m	≤6 weeks	≥3 sessions/week	Adult	HIFT
Ileri and Müniroglu, 2026	COD/agility / soccer-specific speed	No linear sprint	≤6 weeks	≥3 sessions/week	Youth	Functional strength training
Philip et al., 2022	Linear sprint + COD/agility	30 m	≥8 weeks	≥3 sessions/week	Mixed young adult	Multicomponent functional training
Turna and Alp, 2020	Linear sprint only	30 m	≥8 weeks	<3 sessions/week	Adult	Multicomponent functional training
Zhao et al., 2026	COD/agility only	No linear sprint	≥8 weeks	≥3 sessions/week	Youth / prepubescent	FMS-based functional training

**Table 5:** Results of the exploratory subgroup analyses.

Outcome	Moderator	Subgroup	k	Participants, n (FT/CON)	Hedges' g (95% CI)	p value	I² (%)	p for subgroup difference
COD	Intervention duration	≤6 weeks	4	106 (53/53)	-0.54 [-0.93, -0.15]	0.007	0	0.48
		≥8 weeks	5	134 (73/61)	-0.91 [-1.87, 0.04]	0.06	84	
	Weekly frequency	≥3 sessions/week	6	169 (85/84)	-0.97 [-1.68, -0.27]	0.007	78	0.09
		<3 sessions/week	3	71 (41/30)	-0.25 [-0.72, 0.23]	0.32	0	
Linear sprint	Intervention duration	≤6 weeks	3	76 (38/38)	-1.12 [-2.48, 0.25]	0.11	86	0.81
		≥8 weeks	4	88 (45/43)	-0.94 [-1.39, -0.50]	<0.0001	0	
	Weekly frequency	≥3 sessions/week	4	103 (52/51)	-1.10 [-2.04, -0.15]	0.02	79	0.67
		<3 sessions/week	3	61 (31/30)	-0.86 [-1.40, -0.33]	0.002	0	
	Sprint distance	20 m	3	62 (32/30)	-0.61 [-1.15, -0.07]	0.03	8	0.20
		30 m	4	102 (51/51)	-1.26 [-2.09, -0.44]	0.003	71	

COD, change of direction; FT, functional training; CON, control; CI, confidence interval; k, number of studies. Negative effect sizes favor functional training. The final-column p values are from tests for subgroup differences. Weekly frequency was categorized as ≥3 versus <3 sessions/week to match the subgroup forest plots. All subgroup analyses were exploratory.

### 3.6 Sensitivity analysis and publication bias

The leave-one-out sensitivity analyses are presented in **Supplementary Figures S2A, B**. For pre-planned change-of-direction (COD) performance, the pooled effect remained statistically significant after sequential exclusion of each individual study, with Hedges’ g ranging from −0.81 to −0.49. Exclusion of Zhao et al. produced the smallest pooled effect (g = −0.49, 95% CI −0.76 to −0.21), indicating that this study influenced the magnitude of the pooled estimate, although the direction and statistical significance of the overall effect remained unchanged. For linear sprint performance, the pooled effect also remained statistically significant after exclusion of each individual study, with Hedges’ g ranging from −1.13 to −0.75. Exclusion of Doungchan et al. produced the greatest attenuation of the pooled effect (g = −0.75, 95% CI −1.09 to −0.40), but did not alter the overall conclusion.

An additional outcome-selection sensitivity analysis was conducted for linear sprint performance because Erol et al. reported both eligible 10-m and 30-m sprint outcomes. Replacing the 30-m outcome used in the primary analysis with the corresponding 10-m outcome slightly attenuated the pooled effect, which remained statistically significant and favored functional training (Hedges’ g = −0.93, 95% CI −1.52 to −0.34; p = 0.002; τ^2^ = 0.43; I^2^ = 68%). Thus, the direction and statistical significance of the primary sprint finding were robust to the choice of sprint distance from this study. The corresponding forest plot is provided in **Supplementary Figure S3**.

Funnel plots and statistical assessments of small-study effects are presented in **Supplementary Figures S4A, B**. For pre-planned COD performance, visual inspection suggested some funnel-plot asymmetry; however, neither Egger’s regression test (p = 0.112) nor Begg’s rank-correlation test (p = 0.917) provided statistically significant evidence of small-study effects. For linear sprint performance, some asymmetry was also apparent on visual inspection, although neither Egger’s test (p = 0.068) nor Begg’s test (p = 0.072) reached statistical significance.

Given the small number of studies contributing to the COD (k = 9) and sprint (k = 7) analyses, funnel plots and formal tests for small-study effects were interpreted cautiously because of their limited statistical power. Accordingly, the absence of statistically significant tests should not be interpreted as evidence that publication bias was absent.

## 4 Discussion

This systematic review and meta-analysis synthesized evidence from 10 randomized controlled trials examining the effects of functional training on linear sprint and pre-planned change-of-direction (COD) performance in soccer players. The principal findings indicated that functional training was associated with favorable pooled effects on both time-based performance outcomes, reflected by shorter completion times in linear sprint and COD tests. Exploratory subgroup analyses generally yielded effect estimates favoring functional training across different intervention durations, weekly training frequencies, and sprint distances. However, formal tests for subgroup differences did not identify a clear or consistent pattern of effect modification. Consequently, the current evidence does not allow definitive conclusions regarding the intervention duration, training frequency, or sprint distance associated with greater adaptations.

### 4.1 Effects on pre-planned change-of-direction performance

The observed improvements in change-of-direction (COD) performance may reflect integrated neuromuscular adaptations rather than gains in muscular strength alone (Kambitta Valappil et al., 2026). Successful COD actions require players to rapidly decelerate while moving at high speed, lower their center of mass, reposition the body, and coordinate force production across the hip, knee, and ankle joints during braking, directional reorientation, and subsequent reacceleration (Dos'Santos et al., 2018; Marvin et al., 2024; Singh et al., 2025). Consequently, COD performance depends not only on lower-limb strength but also on intersegmental coordination, dynamic balance, trunk control, joint positioning, lower-limb biomechanics, and the effective transmission of force through the kinetic chain (Singh et al., 2025; Kambitta Valappil et al., 2026). he braking and redirection phases are particularly dependent on the capacity to absorb and control horizontal momentum through eccentric force production, followed by the rapid generation of propulsive force to accelerate in the new direction (Liu et al., 2020; Xiao et al., 2021).

Functional training commonly incorporates multijoint, multiplanar, and whole-body movement patterns targeting core stability, unilateral support, dynamic balance, lower-limb strength, movement control, and neuromuscular coordination (Xiao et al., 2021). Such training stimuli may enhance proprioceptive function, postural control, intermuscular coordination, and force transmission during COD tasks, thereby improving movement efficiency and reducing test-completion time (Nawed et al., 2018). Compared with training approaches that primarily isolate individual muscles or physical qualities, functional training may more closely reproduce the integrated movement demands of soccer, including rapid stopping, turning, cutting, evading opponents, and defensive tracking. This task-specific integration may partly explain the favorable pooled effect observed for COD performance. Nevertheless, these mechanisms should be regarded as plausible interpretations rather than directly established mediators, because most included studies did not concurrently assess biomechanical, neuromuscular, or physiological adaptations.

The pooled COD estimate should nevertheless be interpreted with some caution because Zhao et al. exerted a substantial influence on both the magnitude of the pooled effect and the observed heterogeneity. Although exclusion of this study did not alter the direction or statistical significance of the overall finding, the pooled effect was attenuated from g = −0.72 to −0.49 and heterogeneity decreased from I^2^ = 71% to 0%. Thus, the evidence appears robust with respect to the direction of the effect, but less robust with respect to its precise magnitude and the extent of between-study heterogeneity.

### 4.2 Effects on linear sprint performance

The present findings indicate that functional training was associated with improved linear sprint performance in soccer players, as reflected by shorter post-intervention sprint times in the functional training groups. The pooled standardized effect favored functional training; however, its magnitude should not be equated directly with practical or match-performance significance. Nevertheless, the magnitude of the observed effects varied across studies, indicating that the response may depend on the specific intervention content, training dose, participant characteristics, and sprint distance assessed.

Linear sprint performance is a complex motor ability determined by the interaction of neuromuscular capacity, biomechanical characteristics, and running technique (Mero et al., 1992). During short-distance acceleration, players must generate substantial ground-reaction forces within brief ground-contact periods and orient these forces effectively in the horizontal direction to increase the forward velocity of the body’s center of mass (Kugler and Janshen, 2010; Lockie et al., 2011). Sprint performance therefore depends not only on lower-limb strength and power but also on the rate of force development, coordinated extension of the hip, knee, and ankle joints, unilateral support stability, trunk postural control, and the effective coordination of stride frequency and stride length (Morin et al., 2011; Murata et al., 2018; Nagahara et al., 2018). Functional training commonly uses multijoint, unilateral, and whole-body movement patterns that integrate core stability, lower-limb strength, power, postural control, and neuromuscular coordination. Such training may improve force production by the lower-limb extensor musculature, enhance interjoint coordination, and increase stability during unilateral support, while providing a relatively stable trunk–pelvis platform from which rapid lower-limb extension can occur (Philip et al., 2022; Gürgen and Kilinç Boz, 2023). Several included interventions, including suspension training, bodyweight training, functional strength training, and high-intensity functional training, also incorporated rapid extension, stepping, unilateral loading, and coordinated whole-body actions. These stimuli may contribute to the development of rapid force production and more efficient propulsive mechanics, thereby reducing sprint-completion time (Fayazmilani et al., 2022; Haghighi et al., 2026). However, improvements in sprint performance are unlikely to result solely from gains in strength or stability. Their transfer to sprinting may depend on whether these adaptations enhance the effective application of horizontally oriented force during acceleration (Young, 2006; Seitz et al., 2014).

Functional training may therefore provide a general foundation of strength, stability, and coordination, but its practical transfer to linear sprint performance is likely to be influenced by movement velocity, force orientation, ground-contact duration, external load, and the intention to move at maximal speed. Differences in these training characteristics may partly explain the variation in effect magnitude observed across the included studies.

A similar pattern was observed for linear sprint performance. Doungchan et al. had a marked influence on both the pooled effect magnitude and between-study heterogeneity. Excluding this study attenuated the pooled estimate from g = −1.00 to −0.75 while reducing I^2^ from 61% to 0%, although the direction and statistical significance of the effect remained unchanged. Accordingly, the sprint finding appears robust in direction but more sensitive with respect to effect magnitude and heterogeneity.

Importantly, the magnitude of the standardized pooled effects should not be interpreted as direct evidence of practical or match-performance significance. Because the included studies used heterogeneous sprint and COD tests, and generally did not report outcome-specific measurement error or smallest worthwhile change thresholds, the present meta-analysis cannot determine whether the observed improvements exceeded typical measurement variability or represented practically meaningful changes in field performance. Accordingly, the pooled Hedges’ g values should be interpreted as standardized between-group effects rather than direct indicators of meaningful performance enhancement in competitive soccer.

### 4.3 Interpretation of subgroup analyses

Exploratory subgroup analyses were conducted according to intervention duration, weekly training frequency, and sprint distance. Although most subgroup-specific point estimates favored functional training, formal tests for subgroup differences did not provide clear evidence that the magnitude of the effect varied across these factors. Accordingly, the present findings do not establish that any particular training frequency, intervention duration, or sprint distance is associated with greater benefits. Conversely, the absence of statistically significant subgroup differences should not be interpreted as evidence that the effects were equivalent across conditions. The small number of studies within each subgroup, together with the wide confidence intervals observed in several analyses, likely limited the statistical power to detect genuine effect modification. These findings should therefore be considered hypothesis-generating rather than directly translated into definitive training prescriptions (Sun et al., 2010; Richardson et al., 2019).

For linear sprint performance, the pooled point estimate was numerically larger in the 30-m subgroup than in the 20-m subgroup, although the formal test for subgroup differences did not indicate a clear difference between distances. The 20-m sprint primarily reflects the start and early-to-middle acceleration phases, whereas the 30-m sprint additionally captures later acceleration and, in some players, performance approaching maximal running velocity. These phases impose partly distinct demands on body positioning, stride-length and stride-frequency regulation, ground-contact characteristics, and force orientation. Functional training could therefore influence 20- and 30-m sprint performance through partially different neuromuscular and biomechanical pathways (Bellon et al., 2019).Nevertheless, the larger point estimate for the 30-m subgroup should not be interpreted as evidence that functional training is more effective over this distance.

The distance-specific findings should also be considered within the context of soccer match demands. Sprint exposure, available running space, and tactical responsibilities may differ according to playing position; some players may perform short accelerations more frequently, whereas others may encounter more opportunities for longer continuous sprints (Oliva-Lozano et al., 2020; Oliva-Lozano et al., 2023).However, the included studies generally did not report intervention effects separately by playing position or examine interactions between positional role and sprint distance. Position-specific demands therefore represent a plausible contextual consideration, but they cannot explain the numerically larger effect observed in the 30-m subgroup. Consistent with this uncertainty, Keiner et al. did not observe an improvement in 20-m sprint performance following a functional training program based predominantly on elastic resistance, bodyweight, and TRX exercises in highly trained youth soccer players, suggesting that baseline performance level, training load, and movement specificity may jointly influence the transfer of functional training to sprint performance (Keiner et al., 2022).

With respect to weekly training frequency, the subgroups completing at least three sessions per week showed numerically larger favorable effects for both linear sprint and COD outcomes. More frequent exposure may provide greater opportunities to reinforce postural control, force production, and multijoint coordination. However, the available evidence does not demonstrate that three or more weekly sessions are superior to lower-frequency programs (Bashir et al., 2022).Training frequency may also be confounded by other program characteristics, including total training volume, the continuity of progression, session supervision, and the integration of functional training with regular soccer practice (Grgic et al., 2018; Cuthbert et al., 2021). Frequency alone should therefore not be regarded as the sole determinant of the observed differences.

Intervention-duration analyses did not reveal a consistent pattern whereby longer programs produced greater improvements. For linear sprint performance, the pooled effect was more clearly favorable among interventions lasting at least eight weeks. In contrast, interventions lasting six weeks or less also produced favorable effects on COD performance and showed relatively limited within-subgroup heterogeneity. Longer interventions may allow strength, movement control, and neuromuscular coordination to develop progressively, but their effectiveness ultimately depends on whether the program continues to provide a sufficient and progressively challenging stimulus (Kraemer and Ratamess, 2004; Cormie et al., 2011). Extending the intervention period may offer little additional benefit when exercise selection remains unchanged, external loading is insufficient, or progression is absent. Conversely, a well-designed short-term program may elicit relatively rapid improvements through early neural adaptations, enhanced movement proficiency, and increased familiarity with the training tasks (Sander et al., 2013).

Importantly, weekly frequency and intervention duration provide only crude indicators of functional training dose. Movement velocity, external load, number of sets and repetitions, recovery intervals, intent to move rapidly, progression strategy, and the manner in which functional training is combined with routine soccer practice may all influence training adaptations (American College of Sports Medicine, 2009).

These variables were incompletely reported across the included studies, limiting the identification of an optimal training dose (Slade et al., 2016).Within the ranges examined—6–12 weeks and two to three sessions per week—the pooled findings suggest that functional training may benefit both linear sprint and pre-planned COD performance. However, the available evidence is insufficient to determine an optimal weekly frequency, intervention duration, or distance-specific advantage.

### 4.4 Practical applications

Structured functional training may be considered a complementary component of soccer-specific physical preparation for improving linear sprint and pre-planned change-of-direction (COD) performance. Most interventions in the included studies lasted 6–12 weeks and were performed two to three times per week; however, these characteristics should not be interpreted as an optimal training prescription. Program design may progress from foundational stability and movement-control exercises toward more dynamic tasks involving unilateral support, rapid deceleration, directional reorientation, and reacceleration, with load, movement velocity, and complexity adjusted to the player’s training status.

Functional training should complement rather than replace dedicated sprint or COD training. Because the synthesized outcomes were limited to pre-planned movement tasks, improvements should not be assumed to transfer directly to reactive agility or match performance. Recommendations concerning externally triggered agility tasks should therefore be regarded as broader evidence-informed considerations rather than conclusions established by the present meta-analysis. Coaches may individualize the use of functional training according to age, competitive level, training history, and seasonal load, while recognizing the limited evidence base and uncertainty regarding optimal training dose.

### 4.5 Limitations and future recommendations

Several limitations should be considered when interpreting the present findings. First, the available evidence base was small, and several subgroup analyses included only a few studies with wide confidence intervals. Consequently, the absence of significant between-subgroup differences should not be interpreted as evidence that intervention duration, weekly training frequency, or sprint distance does not influence training adaptations. The pooled estimates were also sensitive in magnitude to individual studies and outcome selection. For example, replacing the 30-m sprint outcome from Erol et al. with the corresponding 10-m outcome slightly attenuated the pooled sprint effect, while heterogeneity increased from I^2^ = 61% to 68%; however, the direction and statistical significance of the effect remained unchanged. These findings support robustness of the overall direction of effect but greater uncertainty regarding its precise magnitude.

Second, functional training was implemented heterogeneously across studies, including suspension training, bodyweight training, functional strength training, high-intensity functional training, and multicomponent programs. Intervention delivery also differed with respect to duration, weekly frequency, exercise selection, loading, movement velocity, recovery, and progression. Reporting of these characteristics was often incomplete, limiting the identification of specific training components responsible for the observed effects and precluding reliable conclusions regarding optimal training dose. Future trials should provide more complete and standardized descriptions of intervention content, progression, adherence, supervision, and external training load.

Third, generalizability is limited by the characteristics of the included populations. Most participants were male, and the samples ranged from children and adolescents to amateur and professional adult players. Differences in age, biological maturation, competitive level, training history, and positional demands may influence responsiveness to functional training, but these factors could not be examined reliably with the available evidence. Because several trials involved youth or recreational players and some comparator groups did not receive an additional volume-matched conditioning intervention, part of the observed benefit may reflect exposure to additional structured training rather than an effect specific to functional training. Further adequately powered trials are needed in female players and in more clearly defined age and competitive-level groups.

Finally, the outcome measures impose important limits on practical interpretation. Although several studies labeled the 505, T-test, Arrowhead, and zig-zag tests as agility measures, the synthesized outcomes predominantly represented pre-planned COD tasks and did not capture perceptual, anticipatory, or decision-making components. Improvements should therefore not be interpreted as direct evidence of enhanced reactive agility or match performance. In addition, because the included studies used heterogeneous sprint and COD tests and rarely reported measurement error or smallest worthwhile change thresholds, the standardized pooled effects cannot establish whether the observed improvements were practically meaningful. The assessment of small-study effects was also limited by the small number of studies, and non-significant Egger’s and Begg’s tests should not be interpreted as evidence that publication bias was absent. Future studies should incorporate standardized performance measures, reactive-agility assessments, and match-relevant outcomes to better establish the practical relevance of training adaptations.

## 5 Conclusions

This systematic review and meta-analysis of 10 randomized controlled trials suggests that, compared with usual soccer training, no additional training, or other non-functional exercise conditions, structured functional training may improve linear sprint and pre-planned change-of-direction (COD) performance in soccer players.

The pooled standardized effects were statistically significant for both outcomes; however, these estimates should not be interpreted as direct evidence of practically meaningful or match-performance benefits, and their magnitude varied across studies according to differences in intervention content, participant characteristics, comparator conditions, outcome-assessment methods, and program implementation. The observed improvements may reflect the combined effects of functional training on core stability, lower-limb strength, dynamic balance, postural control, braking capacity, and multijoint neuromuscular coordination, which may collectively enhance force transmission and movement control during sprinting and directional changes. Functional training may therefore be incorporated as a complementary component of soccer-specific physical preparation.

Nevertheless, it should not be considered a substitute for dedicated sprint training, COD technique training, or reactive-agility practice.

Most available interventions lasted 6–12 weeks and were performed two or three times per week. However, subgroup comparisons did not identify clear moderating effects of intervention duration, weekly training frequency, or sprint distance; therefore, these ranges should not be interpreted as an optimal training prescription. Given the small evidence base, limited overall sample size, predominance of male participants, substantial intervention and outcome heterogeneity, incomplete methodological reporting, and very low certainty of evidence, the findings should be interpreted cautiously.

Future adequately powered randomized controlled trials should use transparent and reproducible training prescriptions and examine female players, different age and competitive-level groups, reactive agility, match-related performance, and the long-term retention of training adaptations.

## Data Availability

The original contributions presented in the study are included in the article/**Supplementary Material**. Further inquiries can be directed to the corresponding author.
