# Supplementary material for: Effects of functional training on linear sprint and change-of-direction performance in soccer players: a systematic review and meta-analysis
Source: Front Physiol. 2026 Sep 29;17:1951674. doi: 10.3389/fphys.2026.1951674 (PMC13657990; doi:10.3389/fphys.2026.1951674)
Supplement: Supplementary file 1 [file DataSheet1.docx]

**Supplementary Figures**

*Effects of Functional Training on Linear Sprint and Change-of-Direction Performance in Soccer Players: A Systematic Review and Meta-Analysis*

**Supplementary Figure S1. Exploratory subgroup analyses**

Forest plots for exploratory subgroup analyses. Negative effect sizes favor functional training. Subgroup comparisons were treated as exploratory and hypothesis-generating.

**S1A. COD — intervention duration (≤6 vs. ≥8 weeks)**

**S1B. COD — weekly training frequency (≥3 vs. <3 sessions/week)**

**S1C. Linear sprint — intervention duration (≤6 vs. ≥8 weeks)**

**S1D. Linear sprint — weekly training frequency (≥3 vs. <3 sessions/week)**

**S1E. Linear sprint — sprint distance (20 m vs. 30 m)**

**Supplementary Figure S2. Leave-one-out sensitivity analyses**

Leave-one-out sensitivity analyses for the primary meta-analyses. Each estimate represents the pooled effect after sequential exclusion of one study.

**S2A. COD — leave-one-out sensitivity analysis**

**S2B. Linear sprint — leave-one-out sensitivity analysis**

**Supplementary Figure S3. Outcome-selection sensitivity analysis for linear sprint performance**

Sensitivity analysis replacing the 30-m sprint outcome from Erol et al. with the corresponding 10-m sprint outcome while leaving all other study data unchanged.

**S3. Linear sprint — Erol et al. 10-m outcome replacing the 30-m outcome**

**Supplementary Figure S4. Funnel plots for small-study effects**

Funnel plots for the primary meta-analyses. Because fewer than 10 studies contributed to each outcome, visual and statistical assessments of asymmetry should be interpreted cautiously.

**S4A. COD — funnel plot**

**S4B. Linear sprint — funnel plot**

**Abbreviations:** COD, change of direction.
