# Supplementary material for: Effects of functional training on linear sprint and change-of-direction performance in soccer players: a systematic review and meta-analysis
Source: Front Physiol. 2026 Sep 29;17:1951674. doi: 10.3389/fphys.2026.1951674 (PMC13657990; doi:10.3389/fphys.2026.1951674)
Supplement: Supplementary file 3 [file Table2.docx]

Supplementary Table S2. Complete Search Strategies for All Electronic Databases

**Search framework.** The search combined three concepts: (1) soccer players, (2) functional training, and (3) linear sprint or pre-planned change-of-direction performance. All databases were searched from inception to 6 July 2026. No publication-year restriction or study-design filter was applied. English-language eligibility was assessed during study selection. Database-specific controlled vocabulary, field codes, phrase searching, truncation, and proximity operators were used where available.

# 1. PubMed

| **Database/platform** | National Library of Medicine (PubMed) | **Search date** | 6 July 2026 |
| --- | --- | --- | --- |
| **Coverage** | Database inception to 6 July 2026 | **Records retrieved** | 103 |
| **Concepts combined** | Soccer AND functional training AND sprint/COD | **Final set** | #4 |
| **Filters/limits** | None. No date, language, article-type, or randomized-trial filter was applied in the database query. | | |

| **Set** | **Exact search statement** |
| --- | --- |
| **#1** | "Soccer"[Mesh] OR soccer[Title/Abstract] OR football[Title/Abstract] OR footballer*[Title/Abstract] OR "soccer player"[Title/Abstract] OR "soccer players"[Title/Abstract] OR "football player"[Title/Abstract] OR "football players"[Title/Abstract] |
| **#2** | "functional training"[Title/Abstract] OR "functional exercise"[Title/Abstract] OR "functional exercises"[Title/Abstract] OR "functional strength training"[Title/Abstract] OR "core stability training"[Title/Abstract] OR "core training"[Title/Abstract] OR "suspension training"[Title/Abstract] OR TRX[Title/Abstract] OR "bodyweight training"[Title/Abstract] OR "body weight training"[Title/Abstract] OR "functional neuromuscular training"[Title/Abstract] OR "soccer-specific functional training"[Title/Abstract] OR "football-specific functional training"[Title/Abstract] OR "high-intensity functional training"[Title/Abstract] OR HIFT[Title/Abstract] |
| **#3** | sprint*[Title/Abstract] OR "linear sprint"[Title/Abstract] OR speed[Title/Abstract] OR acceleration[Title/Abstract] OR "change of direction"[Title/Abstract] OR "change-of-direction"[Title/Abstract] OR COD[Title/Abstract] OR agility[Title/Abstract] OR "directional change"[Title/Abstract] |
| **#4** | #1 AND #2 AND #3 |

# 2. Web of Science Core Collection

| **Database/platform** | Clarivate Web of Science Core Collection | **Search date** | 6 July 2026 |
| --- | --- | --- | --- |
| **Coverage** | All years available to 6 July 2026 | **Records retrieved** | 256 |
| **Concepts combined** | Soccer AND functional training AND sprint/COD | **Final set** | #4 |
| **Filters/limits** | Timespan: All years. Indexes: Web of Science Core Collection. No document-type, language, or study-design filter. | | |

| **Set** | **Exact search statement** |
| --- | --- |
| **#1** | TS=(soccer OR football OR footballer* OR "soccer player*" OR "football player*") |
| **#2** | TS=("functional training" OR "functional exercise*" OR "functional strength training" OR "core stability training" OR "core training" OR "suspension training" OR TRX OR "bodyweight training" OR "body weight training" OR "functional neuromuscular training" OR "soccer-specific functional training" OR "football-specific functional training" OR "high-intensity functional training" OR HIFT) |
| **#3** | TS=(sprint* OR "linear sprint" OR speed OR acceleration OR "change of direction" OR "change-of-direction" OR COD OR agility OR "directional change") |
| **#4** | #1 AND #2 AND #3 |

# 3. Embase

| **Database/platform** | Elsevier Embase.com | **Search date** | 6 July 2026 |
| --- | --- | --- | --- |
| **Coverage** | Database inception to 6 July 2026 | **Records retrieved** | 25 |
| **Concepts combined** | Soccer AND functional training AND sprint/COD | **Final set** | #4 |
| **Filters/limits** | No publication-year, language, document-type, or study-design filter. | | |

| **Set** | **Exact search statement** |
| --- | --- |
| **#1** | 'soccer'/exp OR soccer:ti,ab,kw OR football:ti,ab,kw OR footballer*:ti,ab,kw OR (soccer NEAR/2 player*):ti,ab,kw OR (football NEAR/2 player*):ti,ab,kw |
| **#2** | 'functional training':ti,ab,kw OR 'functional exercise':ti,ab,kw OR 'functional exercises':ti,ab,kw OR 'functional strength training':ti,ab,kw OR 'core stability training':ti,ab,kw OR 'core training':ti,ab,kw OR 'suspension training':ti,ab,kw OR trx:ti,ab,kw OR 'bodyweight training':ti,ab,kw OR 'body weight training':ti,ab,kw OR 'functional neuromuscular training':ti,ab,kw OR 'soccer-specific functional training':ti,ab,kw OR 'football-specific functional training':ti,ab,kw OR 'high-intensity functional training':ti,ab,kw OR hift:ti,ab,kw |
| **#3** | sprint*:ti,ab,kw OR 'linear sprint':ti,ab,kw OR speed:ti,ab,kw OR acceleration:ti,ab,kw OR 'change of direction':ti,ab,kw OR 'change-of-direction':ti,ab,kw OR cod:ti,ab,kw OR agility:ti,ab,kw OR 'directional change':ti,ab,kw |
| **#4** | #1 AND #2 AND #3 |

# 4. SPORTDiscus

| **Database/platform** | SPORTDiscus via EBSCOhost | **Search date** | 6 July 2026 |
| --- | --- | --- | --- |
| **Coverage** | Database inception to 6 July 2026 | **Records retrieved** | 68 |
| **Concepts combined** | Soccer AND functional training AND sprint/COD | **Final set** | S4 |
| **Filters/limits** | No publication-year, language, publication-type, or study-design limiter. | | |

| **Set** | **Exact search statement** |
| --- | --- |
| **S1** | TX (soccer OR football OR footballer* OR "soccer player*" OR "football player*") |
| **S2** | TX ("functional training" OR "functional exercise*" OR "functional strength training" OR "core stability training" OR "core training" OR "suspension training" OR TRX OR "bodyweight training" OR "body weight training" OR "functional neuromuscular training" OR "soccer-specific functional training" OR "football-specific functional training" OR "high-intensity functional training" OR HIFT) |
| **S3** | TX (sprint* OR "linear sprint" OR speed OR acceleration OR "change of direction" OR "change-of-direction" OR COD OR agility OR "directional change") |
| **S4** | S1 AND S2 AND S3 |

# 5. Scopus

| **Database/platform** | Elsevier Scopus | **Search date** | 6 July 2026 |
| --- | --- | --- | --- |
| **Coverage** | Database inception to 6 July 2026 | **Records retrieved** | 51 |
| **Concepts combined** | Soccer AND functional training AND sprint/COD | **Final set** | #4 |
| **Filters/limits** | No publication-year, language, document-type, or study-design filter. | | |

| **Set** | **Exact search statement** |
| --- | --- |
| **#1** | TITLE-ABS-KEY (soccer OR football OR footballer* OR "soccer player*" OR "football player*") |
| **#2** | TITLE-ABS-KEY ("functional training" OR "functional exercise*" OR "functional strength training" OR "core stability training" OR "core training" OR "suspension training" OR TRX OR "bodyweight training" OR "body weight training" OR "functional neuromuscular training" OR "soccer-specific functional training" OR "football-specific functional training" OR "high-intensity functional training" OR HIFT) |
| **#3** | TITLE-ABS-KEY (sprint* OR "linear sprint" OR speed OR acceleration OR "change of direction" OR "change-of-direction" OR COD OR agility OR "directional change") |
| **#4** | #1 AND #2 AND #3 |

# 6. Cochrane Library

| **Database/platform** | Cochrane Library (Wiley) | **Search date** | 6 July 2026 |
| --- | --- | --- | --- |
| **Coverage** | Database inception to 6 July 2026 | **Records retrieved** | 239 |
| **Concepts combined** | Soccer AND functional training AND sprint/COD | **Final set** | #6 |
| **Filters/limits** | No publication-year or language restriction. All matching Cochrane Library records were exported and screened. | | |

| **Set** | **Exact search statement** |
| --- | --- |
| **#1** | MeSH descriptor: [Soccer] explode all trees |
| **#2** | (soccer OR football OR footballer* OR "soccer player*" OR "football player*"):ti,ab,kw |
| **#3** | #1 OR #2 |
| **#4** | ("functional training" OR "functional exercise*" OR "functional strength training" OR "core stability training" OR "core training" OR "suspension training" OR TRX OR "bodyweight training" OR "body weight training" OR "functional neuromuscular training" OR "soccer-specific functional training" OR "football-specific functional training" OR "high-intensity functional training" OR HIFT):ti,ab,kw |
| **#5** | (sprint* OR "linear sprint" OR speed OR acceleration OR "change of direction" OR "change-of-direction" OR COD OR agility OR "directional change"):ti,ab,kw |
| **#6** | #3 AND #4 AND #5 |

# 7. Additional identification methods

| **Source** | **Method** | **Date consulted** | **Records identified** |
| --- | --- | --- | --- |
| Reference lists | Manual screening of the reference lists of all included studies and relevant systematic reviews. | 6 July 2026 | 8 |

**Abbreviations:** AB, abstract; COD, change of direction; HIFT, high-intensity functional training; KW, keyword; MeSH, Medical Subject Headings; TI, title; TS, Topic; TX, all-text field; TRX, Total Resistance Exercises.
