# Supplementary material for: Effects of functional training on linear sprint and change-of-direction performance in soccer players: a systematic review and meta-analysis
Source: Front Physiol. 2026 Sep 29;17:1951674. doi: 10.3389/fphys.2026.1951674 (PMC13657990; doi:10.3389/fphys.2026.1951674)
Supplement: Supplementary file 4 [file Table3.docx]

**Supplementary Table S3. GRADE summary of findings for linear sprint and change-of-direction performance**

| **Outcome** | **Participants (studies)** | **Pooled effect** | **Risk of bias** | **Inconsistency** | **Indirectness** | **Imprecision** | **Publication bias** | **Certainty of evidence** |
| --- | --- | --- | --- | --- | --- | --- | --- | --- |
| Pre-planned COD performance | 240 (9 RCTs) | Hedges’ g −0.72 (95% CI −1.23 to −0.22) | Seriousᵃ | Seriousᵇ | Not serious | Seriousᶜ | Not downgradedᵈ | ⊕◯◯◯ Very low |
| Linear sprint performance | 164 (7 RCTs) | Hedges’ g −1.00 (95% CI −1.54 to −0.46) | Seriousᵃ | Seriousᵇ | Not serious | Seriousᶜ | Not downgradedᵈ | ⊕◯◯◯ Very low |

**Note.** CI, confidence interval; COD, pre-planned change of direction; RCT, randomized controlled trial. Hedges’ g is a small-sample corrected standardized mean difference. Negative values favor functional training because lower completion times indicate better performance.

**ᵃ** Downgraded one level for risk of bias because all contributing outcome results were judged as having some concerns in the RoB 2 assessment, mainly owing to incomplete reporting of randomization/concealment, outcome-measurement procedures, or selective-reporting safeguards.

**ᵇ** Downgraded one level for inconsistency because substantial heterogeneity remained in the primary analyses (I² = 71% for pre-planned COD and I² = 61% for linear sprint), and the magnitude of the pooled estimates was sensitive to individual influential studies.

**ᶜ** Downgraded one level for imprecision because the total information size was limited and the 95% CIs, although excluding the null, encompassed meaningfully different standardized effect magnitudes. Outcome-specific measurement-error or smallest-worthwhile-change thresholds were generally unavailable, limiting judgments about practical importance.

**ᵈ** No downgrade was applied for publication bias. Fewer than 10 studies contributed to each outcome, so funnel plots and Egger/Begg tests had limited power; publication and selective-reporting biases therefore cannot be excluded.

**Interpretation.** The certainty of evidence was rated very low for both outcomes. The pooled standardized effects favor functional training, but the true effects may differ substantially from these estimates, and the standardized effect sizes should not be interpreted as direct evidence of practically meaningful or match-performance benefits.
