# Supplementary material for: Effects of functional training on linear sprint and change-of-direction performance in soccer players: a systematic review and meta-analysis
Source: Front Physiol. 2026 Sep 29;17:1951674. doi: 10.3389/fphys.2026.1951674 (PMC13657990; doi:10.3389/fphys.2026.1951674)
Supplement: Supplementary file 5 [file Table4.docx]

**Supplementary Table S4. Detailed RoB 2 judgments and brief supporting rationale**

| **Study** | **Outcome** | **D1** | **D2** | **D3** | **D4** | **D5** | **Overall** | **Brief rationale** |
| --- | --- | --- | --- | --- | --- | --- | --- | --- |
| Atıcı 2025 | Sprint/COD | SC | L | L | L | SC | SC | Concealment unclear; electronic timing; no prospective analysis plan. |
| Boz 2023 | Sprint/COD | SC | L | L | SC | SC | SC | Sequence/concealment unclear; one withdrawal; timing/blinding unclear. |
| Doungchan 2026 | Sprint/COD | L | L | L | SC | SC | SC | Concealed randomization; stopwatch timing; assessor blinding unclear. |
| Erol 2023 | Sprint/COD | SC | L | L | L | SC | SC | Concealment unclear; electronic timing; no prospective analysis plan. |
| Fayazmilani 2022 | COD | SC | L | L | SC | SC | SC | Concealment unclear; manual stopwatch; assessor blinding not reported. |
| Haghighi 2026 | Sprint/COD | L | L | L | SC | SC | SC | Concealed randomization; complete follow-up; stopwatch/unblinded assessment. |
| İleri 2026 | COD | L | L | L | L | SC | SC | Independent randomization; blinded evaluators; photocell timing. |
| Philip 2022 | Sprint/COD | SC | L | L | SC | SC | SC | Sequence/concealment unclear; timing/blinding insufficiently described. |
| Turna 2020 | Sprint | SC | L | L | L | SC | SC | Sequence/concealment unclear; photocell timing; no prospective plan. |
| Zhao 2026 | COD | SC | L | L | L | SC | SC | Concealment unclear; blinded standardized assessment; no prospective plan. |

**Note.** L, low risk of bias; SC, some concerns. D1, randomization process; D2, deviations from intended interventions; D3, missing outcome data; D4, measurement of the outcome; D5, selection of the reported result. All assessments target the effect of assignment to intervention. COD, pre-planned change of direction. No outcome was judged at high risk of bias.
